# Assessing the Impact of Deforestation of the Atlantic Rainforest on Ant-Fruit Interactions: A Field Experiment Using Synthetic Fruits

**DOI:** 10.1371/journal.pone.0090369

**Published:** 2014-02-26

**Authors:** Ana Gabriela D. Bieber, Paulo S. D. Silva, Sebastián F. Sendoya, Paulo S. Oliveira

**Affiliations:** 1 Programa de Pós-Graduação em Ecologia, Instituto de Biologia, Universidade Estadual de Campinas, Campinas SP, Brazil; 2 Laboratório de Biossistemática Animal, Universidade Estadual do Sudoeste da Bahia, Itapetinga BA, Brazil; 3 Departamento de Biologia Animal, Universidade Estadual de Campinas, Campinas SP, Brazil; University of Marburg, Germany

## Abstract

Ants frequently interact with fleshy fruits on the ground of tropical forests. This interaction is regarded as mutualistic because seeds benefit from enhanced germination and dispersal to nutrient-rich microsites, whereas ants benefit from consuming the nutritious pulp/aril. Considering that the process of deforestation affects many attributes of the ecosystem such as species abundance and composition, and interspecific interactions, we asked whether the interaction between ants and fallen fleshy fruits in the Brazilian Atlantic forest differs between human-created fragments and undisturbed forests. We controlled diaspore type and quantity by using synthetic fruits (a plastic ‘seed’ covered by a lipid-rich ‘pulp’), which were comparable to lipid-rich fruits. Eight independent areas (four undisturbed forests, and four disturbed forest fragments) were used in the field experiment, in which we recorded the attracted ant species, ant behaviour, and fruit removal distance. Fruits in undisturbed forest sites attracted a higher number of species than those in disturbed forests. Moreover, the occurrence of large, fruit-carrying ponerine ants (*Pachycondyla*, *Odontomachus*; 1.1 to 1.4 cm) was higher in undisturbed forests. Large species (≥3 mm) of *Pheidole* (Myrmicinae), also able to remove fruits, did not differ between forest types. Following these changes in species occurrence, fruit displacement was more frequent in undisturbed than in disturbed forests. Moreover, displacement distances were also greater in the undisturbed forests. Our data suggest that fallen fleshy fruits interacting with ants face different fates depending on the conservation status of the forest. Together with the severe loss of their primary dispersers in human-disturbed tropical forest sites, vertebrate-dispersed fruits may also be deprived of potential ant-derived benefits in these habitats due to shifts in the composition of interacting ant species. Our data illustrate the use of synthetic fruits to better understand the ecology of ant-fruit interactions in variable ecological settings, including human-disturbed landscapes.

## Introduction

Biodiversity loss through habitat fragmentation and deforestation is directly caused by loss of original habitat, reduction of the remaining area, increased isolation of the remnants, and increased remnant area under edge effects [1], [2]. Moreover, direct alterations in species composition and abundance are frequently related to changes and/or disruptions of ecologically important interactions (e.g. [3], [4], [5]). Particularly, the loss of mutualistic interactions involved in plant reproduction and recruitment may have drastic consequences for ecosystem functioning as a whole [6], [7]. One such important interaction is seed dispersal by animals, mainly vertebrates, which benefits plants either by the escape of density-dependent mortality near the parent tree, the colonization of new habitats, or the directed dispersal to more suitable micro-habitats (see review in [8]). Due to habitat loss and fragmentation, as well as hunting, large vertebrate species (including frugivores) have already disappeared from many small fragments in the tropical region [9], [10]. In the long run, the continual disappearance of vertebrate frugivores can lead to local extinction of certain plant species that depend on animals for seed dispersal ([4], [11], [12] but see [13], [14]).

Although ca. 90% of the plants in Neotropical forests bear vertebrate-dispersed seeds [15], large amounts of fruits can fall intact to the forest floor (spontaneously or dropped by frugivores; [16], [17]), and interactions with some invertebrate groups have been shown to provide dispersal benefits [18]. In the last two decades several studies have documented that ground-dwelling ants can play a key role in the dispersal, germination and, to a lesser degree, establishment of non-myrmecochorous plant species [14], [16], [17], [19], [20]. For instance, whereas seed cleaning (i.e., pulp removal) by ants increases germination success [21], [22], [23], directed seed dispersal to nutrient-rich ant nests improves seedling establishment [17], [24], [25]. Since ants also benefit from consuming the nutritious pulp/aril, this opportunistic interaction is commonly viewed as mutualistic [19].

Due to their smaller body size, smaller home range, and lower trophic position, insect populations are considered less susceptible than vertebrates to the loss of forested area *per se* [2], [26]. Moreover, while vertebrates are directly threatened by hunting [9], the only direct threat imposed by humans to insects is pest control (e.g., leaf-cutter ants in cultivated areas; [27]). Thus, ants and their ecological interactions in general should be considered less threatened by habitat fragmentation [28]. However, it is worth noting that tropical ants are especially sensitive to climate warming, which is normally associated with ongoing global climate change, but may also be linked to alterations in pristine forest habitats following fragmentation [29]. Indeed, no difference was found regarding species richness of myrmecophytes (plants with specialized organs that house ant colonies) and their associated ants between continuous forests and 25-year isolated fragments in the Brazilian Amazon [30]. However, recent studies on the interactions between ants and non-myrmecochorous diaspores in human-disturbed tropical landscapes (such as edge habitats [20], [31], [32] and early secondary forests [33], [34]) have shown that changes in ant species composition can affect ant-induced benefits to seeds (dispersal, seed cleaning).

The Brazilian Atlantic rainforest is considered one of the world's most threatened ecosystems [35]. Habitat loss and other human-associated disturbances (hereafter referred only as deforestation) have increased drastically in the past few decades [36], and only 13% of the original forest remains interspersed in a highly fragmented landscape [37]. In this biome, human-related disturbances have already been shown to affect ground-dwelling ant communities [38], [39], [40]. For example, marked changes in species composition were registered between forest edge and forest core habitats [38], as well as between regenerating secondary forests and primary forests [39]. In addition, particular functional groups of ants (e.g. cryptic species, specialized predators, and climate specialists) were shown to be sensitive to forest size and habitat fragmentation [40]. To our knowledge, however, no study has hitherto compared patterns of ant-fruit interaction between forest fragments and unaltered, continuous tracts of tropical rainforest.

Recent studies have shown that large amounts of the fruit crop produced by tropical trees can reach the forest floor intact (i.e., with the pulp still attached), falling passively or dropped by vertebrate frugivores [16], [17], [25], [41], [42]. Here, we investigate how deforestation of the Atlantic forest affects the interaction between ants and fallen non-myrmecochorous fruits in a fragmented landscape of São Paulo State, SE Brazil. More specifically, the following response variables were compared between disturbed and undisturbed forest sites: richness of ants attending fruits, ant species density *per* station, frequency of particular ant groups, frequency of fruit removal and of pulp removal by ants, and distance of fruit removal. Diaspore type and quantity were controlled by offering synthetic fruits whose size and composition were comparable to fruits of many non-myrmecochorous species found in different physiognomies of the Atlantic rainforest [43], [44], including the current study area [45]. Our experimental study thus simulates primary dispersal by ants of fruits that have fallen passively (i.e., unmanipulated by frugivores) to the forest ground. Under these controlled conditions and based on previous data on ant responses to fragmentation [28], we expected to find similar values at fragments and continuous forests in terms of general ant attendance to ‘fruits’ (i.e., number of ‘fruit’ stations visited and ant species density per station). However, we also expected a shift in ant species composition (e.g., reduced frequency of large ponerines in fragments; [20]), which could lead to a decrease of important ant services to plant diaspores (mostly ‘seed’ removal). Our results show that patterns of ant-fruit interaction are indeed markedly affected by Atlantic rainforest fragmentation, mainly due to a lower occurrence of large, seed-carrying ants in the fragmented areas.

## Materials and Methods

### Ethics Statement

All necessary permits were obtained for the described study, which complied with all relevant regulations. The Brazilian Institute of Environment and Renewable Natural Resources allowed ant samplings in all private and protected forest sites (SISBIO/IBAMA, Permit Number 13666-1 and 13666-2). Moreover, private land owners and the Forestry Institute of São Paulo (Process number 44.174/2007) also authorized our entry in their specific areas.

### Study Site

This study was carried out in the municipalities of Piedade and Tapiraí (23°50′S, 47°20′W) at São Paulo State, Southeast Brazil. Native vegetation is classified as lower montane Atlantic rain forest [46], with altitudes ranging from 750 to 1,000 m a.s.l. The climate is characterized by a warm rainy summer (October to March; accounting for nearly 65% of the annual rainfall) and by the absence of a true dry season during winter (April to September). Monthly mean temperatures vary from 15° to 22°C and rainfall is ca. 1,800 mm yr^−1^ [47].

**Figure 1:**
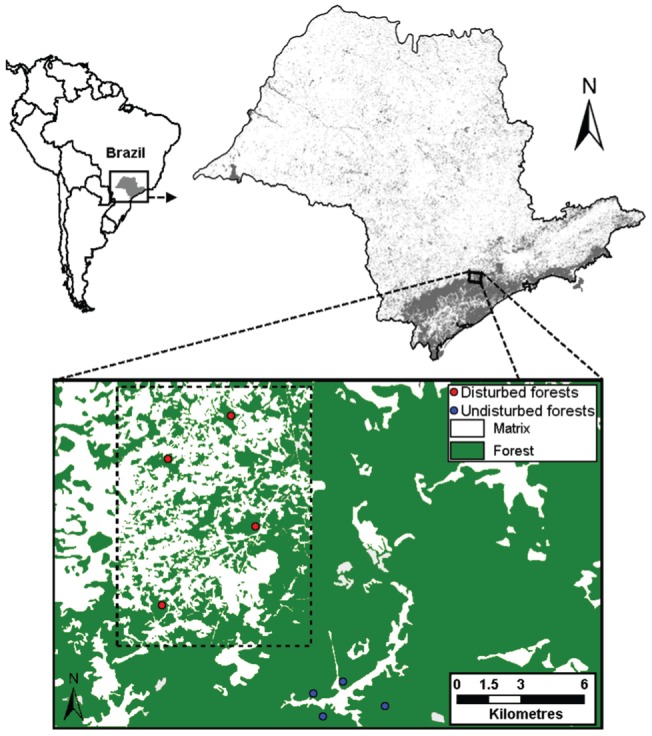
Study area location at the municipalities of Piedade and Tapiraí, São Paulo State, Brazil. The rectangle with dotted borders defines the fragmented disturbed landscape where we selected four forest fragments of ca. 100‘Parque Estadual de Jurupará’. The white area situated within the four continuous forest sites represents the Jurupará Dam. Modified from Banks-Leite *et al.* (2010).

The fragmented landscape presents ca. 50% of remaining forest cover, divided in fragments of secondary forests of various sizes and at various successional stages, from 25 to more than 60 years old [48]. We used four secondary forest fragments (hereafter referred as disturbed forests, DFs) ranging from 91 to 146 ha, which were surrounded mainly by herbaceous cropland, such as ginger and yam, and by pastures [49] (see Figure 1). Located just 5 km from this fragmented landscape, the nearly continuous and undisturbed forest inside the state preserve ‘Parque Estadual de Jurupará’ (PEJU) was used as the control area (hereafter referred as undisturbed forests, UFs). This continuous forest area consists of a 26,000 ha of old-regrowth secondary forests in a late successional stage [49]. Replications for the undisturbed forest were obtained by selecting four areas inside PEJU at least 1.5 km apart from each other, which were considered as spatially independent (for similar approaches, see [49], [50]). Disturbed and undisturbed forest sites are not in the same successional stage in our study landscape, but climatic and edaphic conditions are similar among study sites [48], [49]. This pattern is due to the much older occupancy history of this part of Brazil in comparison with the Amazon forest, and reflects the state of most fragments in the Atlantic forest biome [36], [37].

### Synthetic Fruits

**Figure 2:**
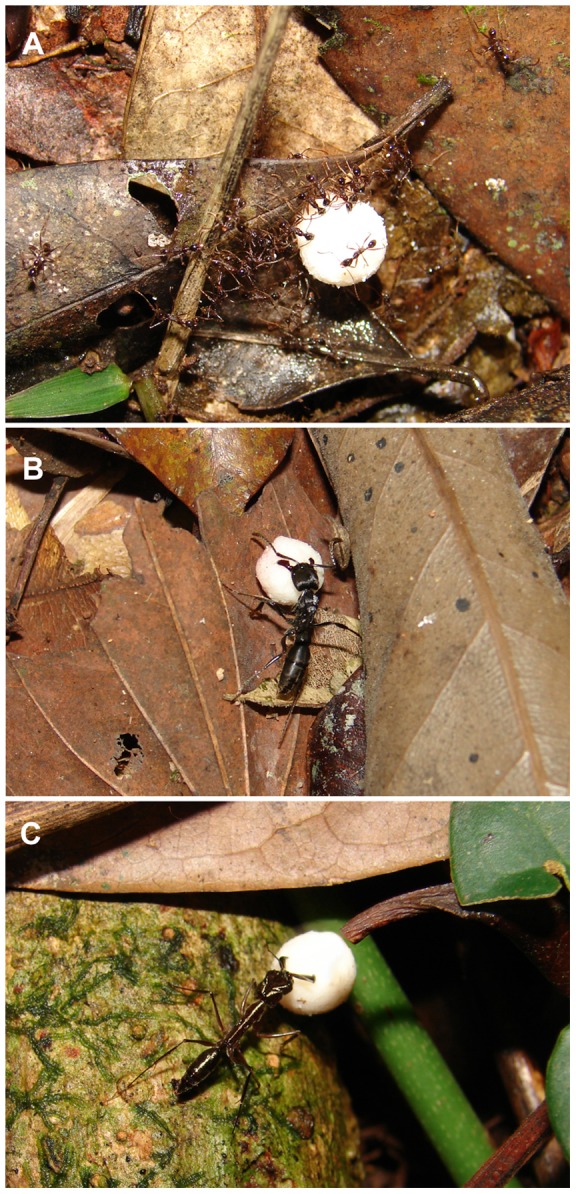
Behavioural interactions between ants and lipid-rich synthetic fruits in the Atlantic forest, Southeast Brazil. (A) Recruited workers of a large *Pheidole* species (≥3 mm) displacing a ‘fruit’; individual workers of (B) *Pachycondyla striata* (∼1.2 cm) and of (C) *Odontomachus chelifer* (∼1.4 cm) carrying a synthetic fruit to the nest. See also Table S1.

The application of artificial models is useful in ecological and behavioural studies when natural models are not available in sufficient numbers to allow experimentation, or when testing a particular hypothesis requires the manipulation of specific traits. For instance, synthetic fruits can be used to investigate how different fruit traits affect response patterns by frugivores [51]. Here, we used synthetic fruits because we needed a large quantity of fallen fleshy fruits in the same condition (i.e., ripeness and handling [52]), and because only very few plant species were found fruiting in the eight study sites simultaneously [45]. Our artificial fruits (hereafter also referred as ‘fruits’) contained a lipid-rich ‘pulp’, since ants show a high preference for lipid-rich plant diaspores [43], [53]. The synthetic pulp recipe was developed by the Institute of Food Technology (ITAL, Campinas, Brazil), in accordance with the chemical composition of fleshy fruits most attractive to ants in Atlantic rainforest [43], [53], and consisted of cotton fat SC (75% of the entire weight), casein (7%), maltodextrin (5%), fructose (4.8%), glucose (4.7%), calcium carbonate (3%), and sucrose (0.5%) (see also [54]). As ‘seeds’ we used red plastic beads of ca. 0.06 g and 3 mm diameter. Each synthetic fruit contained a single ‘seed’ entirely covered by the whitish ‘pulp’, with a total weight of ca. 0.2 g and 8 mm diameter (Figure 2). Final weight and size of synthetic fruits fit between the ‘small’ to ’medium’ size categories (weight 0.05–0.90 g, diameter 5–13 mm) previously adopted in experiments of fruit removal by ants in Atlantic forest [53]. Indeed, a wide spectrum of ant species is capable of removing fruits in these size categories, either as solitary or recruited foragers (see [43], [44]).

### Experimental Design

In each of the eight study areas (four DFs and four UFs), we established thirty sampling stations 10 m apart from each other (to ensure independent discoveries by different ant colonies) along one transect. In the undisturbed forest sites, transects were established at least 300 m from any forest edge to avoid confounding edge affects. In disturbed fragments, since edge effects normally come together with area reduction effects [1], we kept a minimum distance of at least 50 m to the nearest edge. Thus, although this design does not enable the discrimination between edge and area reduction effects, the achieved results should be representative of most fragments in the Brazilian Atlantic Forest, which due to their small size and irregular shape are almost entirely subjected to the influence of edge effects [37]. In each sampling station we deposited on the ground five synthetic fruits on a piece of white filter paper (4×4 cm) to facilitate visualization. ‘Fruits’ were covered with a wire cage (1.5 cm mesh) to exclude vertebrate access (see [53]). The experiment was set at 10∶00 a.m. and ant attendance to ‘fruits’ at sampling stations was checked at 11∶00 a.m., 01∶00 p.m., and 03∶00 p.m. During each sampling, we recorded the attracted ant species and their behaviour toward the ‘fruits’. Ants were collected only if there were more than five individuals at a sampling station (most often). Large non-recruiting ants (*Pachycondyla*, *Odontomachus*) were identified on spot. Also, during each sampling, the location of each removed ‘fruit’ was tagged with wooden sticks to facilitate encounter at the end of the experiment. Stations were checked once again in the following day at 08∶00 a.m. to record the number of synthetic fruits cleaned (i.e., pulp entirely removed) or removed by ants and, when applicable, we measured the removal distance. A ‘fruit’ was considered removed if not found within a 30-cm radius of its original position (*sensu* [16]). Displacement distances were measured only for ‘fruits’ whose final locations were detected, including those found within the 30-cm radius. ‘Fruits’ were considered as cleaned when more than 75% of its ‘pulp’ had been detached by ants after 22 hours of exposure (i.e., until 08∶00 a.m. of the next day).

The eight study sites were sampled on consecutive days (17 to 27 March 2010), under similar weather conditions (sunny to partially cloudy days; no rainfall). Because ant species live in sessile colonies that generally last more than one year [55], we believe that our comparative data on ant activity among forest sites were not biased by any relevant seasonal effect.

### Data Analyses

Our predictor variable for the all statistical analyses was the type of forest (disturbed or undisturbed). Total ant species richness in the different forest sites was compared by estimating species richness using the Jackknife I procedure (see [56]) in the program EstimateS (Version 7.5, R. K. Colwell, http://purl.oclc.org/estimates). Based on the richness estimates and the standard errors, we calculated the corresponding 95% confidence intervals.

To compare species composition among the eight sampling sites, we used a non-metric multidimensional scaling ordination (NMDS), based on the Bray-Curtis index of similarity between studied sites for presence-absence data. This was followed by an Analysis of Similarity (ANOSIM), based on 1000 permutations, for comparing if the two habitat types significantly differ in terms of species composition. Both analyses were performed with the software Primer version 5 (2001; PRIMER-E, Plymouth, UK).

A nested analysis of variance (ANOVA) was used to compare the number of ant species per sampling station between the two forest types, while the study sites were treated as a random factor nested within forest type. Residuals normality and homogeneity of variances were tested previously with Shapiro-Wilk's and Levene's tests, respectively. Sampling stations from which we were unable to collect ant attendants (i.e., ants were very fast at displacing ‘fruits’) were removed from this analysis (only 10 cases removed; *n* = 230 stations). These tests were performed using Statistica version 8.0 (2007; StatSoft Inc., Tulsa, USA).

For several analyses (visitation by ants at the first hour; occurrence of ‘fruit’ removal and cleaning at stations; occurrence of particular ant species groups at stations; and proportion of ‘fruits’ removed per station), we adopted a generalized linear mixed effect model (GLMM) procedure performed with package ‘glmmADMB’ for the software R 2.14.2 [57], considering habitat type as a fixed effect and sites nested within habitat as a random effect. Due to the nature of our response variable (i.e., presence/absence or proportional data), we used a binomial error distribution and a logit link function to fit the model. Categorization of ant groups was determined *a posteriori*, although we expected *a priori* that large ponerine ants would be one of such beneficial groups through due to their recognized ability to remove fruits to greater distances (see [19]). To compare ‘fruit’ displacement distances between continuous and fragmented forest sites, we used only those ‘fruits’ which were displaced at least 1 cm from their stations and whose final location was recorded at the end of the experiment. We also adopted a GLMM procedure and used a gamma distribution to fit the model; the original distance data were Log10 (x+1) transformed to avoid overdispersion problems in model fitting. Significance of the fixed effect for these seven analyses was inferred based on a Wald *Z*-test.

Statistical procedures follows [58] and [59].

## Results

### The Ant Fauna Attracted To Synthetic Fruits

**Figure 3:**
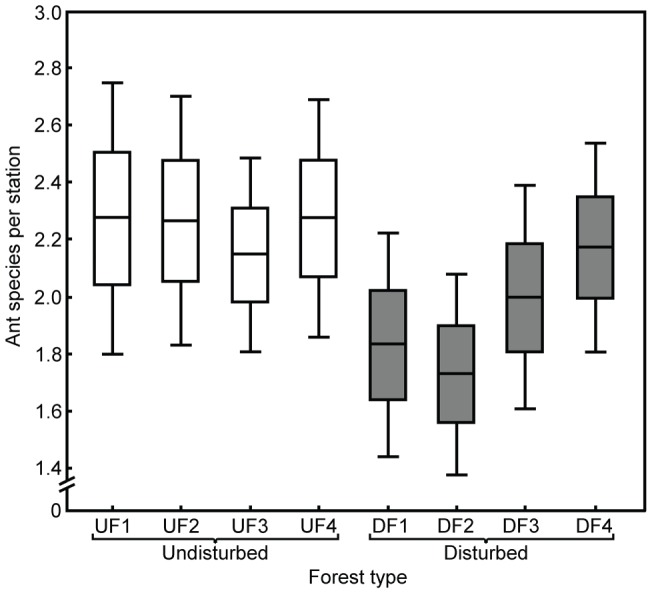
Ant species per station baited with synthetic fruits in undisturbed and disturbed Atlantic forest sites. Thirty stations were sampled per site. Central lines and borders in each box-plot indicate the mean value and the mean ± its standard error; whiskers delimit the range of the 95% confidence interval.

A total of 51 ant species were attracted to the lipid-rich synthetic fruits (see Table S1, Supporting Information). Although other invertebrates such as springtails, crickets, harvestmen, spiders and flies were also occasionally attracted to the ‘fruits’, they neither discouraged approaching ants nor removed themselves any ‘fruit’. Ant richness ranged from 16 to 24 species per forest site (Table S1). Estimated species richness differed among the eight sampled areas; three undisturbed forest areas (UF1, UF2, UF4) and one disturbed fragment site (DF2) presented the highest estimated richness values, whereas fragment DF3 presented the lowest estimated richness (see Figure S1). Moreover, the number of ant species per sampling station varied from none (three stations at disturbed forests) to six (two stations at undisturbed sites). Overall, the mean number of species per station was higher in undisturbed (2.24±0.10; mean ± SE) than in disturbed forest sites (1.94±0.09; F_1, 222_  = 4.89, p<0.03), with no nested effect of the site (F_6, 222_  = 0.52, p = 0.79; Figure 3).

The ant species most frequently recorded at sampling stations were *Pheidole* sp. 3 (in 51 out of 240 stations; six sites), *Solenopsis* sp. 7 (44 stations; all sites), *Pheidole* sp. 1 (39 stations; seven sites), *Pachycondyla striata* (36 stations; seven sites), *Pheidole* sp. 8 and *Wasmannia affinis* (25 stations and seven sites each). The genera *Pheidole* and *Solenopsis* presented the greatest number of species in both habitat types, although the frequency of particular species in these genera differed between disturbed and undisturbed forests (e.g., *Pheidole* sp. 3, *Pheidole* sp. 12, *Solenopsis* sp. 1; see Table S1).

**Figure 4:**
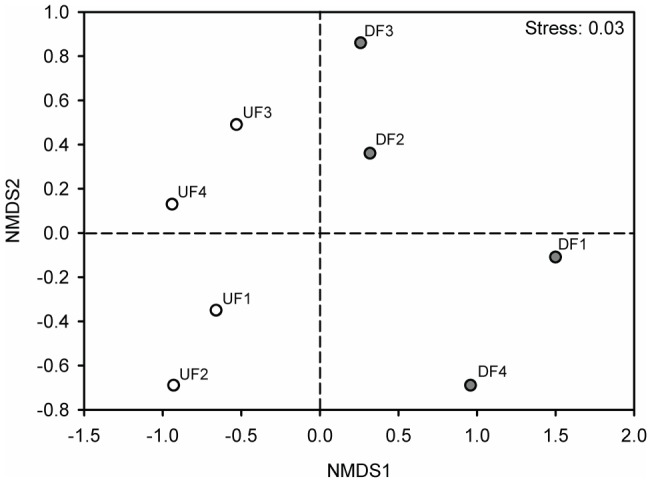
Non-metric multidimensional scaling ordination of the ant community in undisturbed and disturbed Atlantic forest sites. Thirty stations baited with synthetic fruits were sampled in four undisturbed (white circles; UFs) and four disturbed forest sites (gray circles; DFs). Ordination was based on Bray–Curtis index of similarity between studied sites for presence-absence data.

NMDS ordination of the eight studied sites based on species similarity (presence-absence data) partially segregated disturbed and undisturbed forest sites (Figure 4); plot ordination was well supported by a low stress level of 0.03. Indeed, ANOSIM uncovered a significant effect of forest type (Global R = 0.74, p<0.03). Furthermore, with respect to ant species composition, two disturbed fragments (DF2 and DF3) are more similar to the undisturbed forests than to the other two disturbed forest sites (DF1 and DF4; Figure 4).

**Figure 5:**
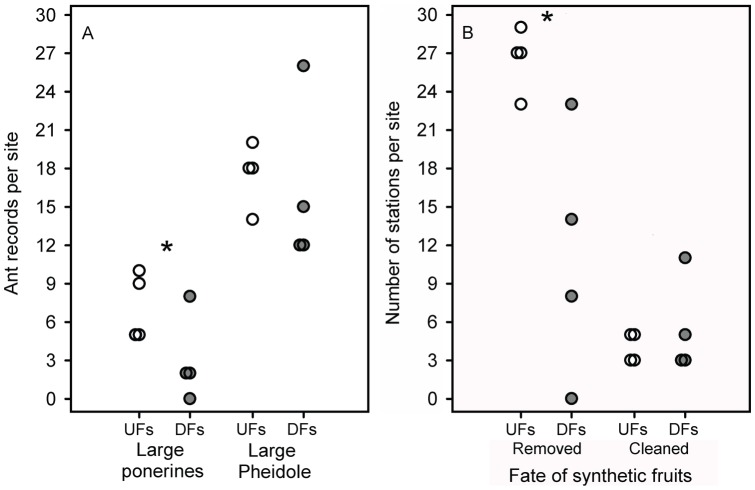
Ant attendance and ant behaviour toward synthetic fruits. (A) Occurrence of particular ant groups and (B) occurrence of beneficial behaviours at sampling stations. Experiments with synthetic fruits were carried out in two diverging forest types in the Atlantic forest, Southeast Brazil, undisturbed (white circles; four sites) and fragmented forest sites (gray circles; four sites). The ant groups (large ponerines and large *Pheidole* spp.) were those whose behaviours were considered as potentially beneficial to ‘seeds’ (either removing the entire ‘fruit’ or cleaning the ‘pulp’ *in situ*) during the 22-hour experiment. The number of stations (y-axis) in (B) corresponds to those stations having at least one of the five seeds either removed or cleaned by ants. Asterisks indicate significant differences (p<0.5) between forest types.

Two large ponerines, *Pachycondyla striata* and *Odontomachus chelifer*, and a few large species of *Pheidole* (Myrmicinae) were amongst the most frequent removers of synthetic fruits (see Figure 2 and Table S1). The ant species most commonly seen cleaning the ‘pulp’ from synthetic fruits were *Megalomyrmex iheringi*, *Solenopsis* sp. 11, and a few *Pheidole* species. However, most ant species (70%), especially the small ones, were neither capable of displacing synthetic fruits nor of entirely detaching the synthetic pulp (see Table S1). Field observations revealed that large ponerines and large *Pheidole* were the ants most likely to provide beneficial services to ‘seeds’ (i.e., dispersal, or ‘pulp’ detaching). *Pachycondyla striata* and *Odontomachus chelifer* were the main removers of synthetic diaspores and their presence was higher in undisturbed forest sites than in disturbed areas (Wald's Z = −2.03, p<0.05; Figure 5A). On the other hand, large species of *Pheidole* (body length ≥3 mm) were frequently seen performing ‘seed’ cleaning on spot and/or ‘fruit’ displacement. This myrmicine group was equally frequent in undisturbed and disturbed forests (Wald's Z = −1.09, p = 0.28; Figure 5A) (Z_adjusted_  = 0.88, p = 0.38) (see Figure 5A and Table S1).

### Ant-Fruit Interactions In Disturbed And Undisturbed Forests

**Figure 6:**
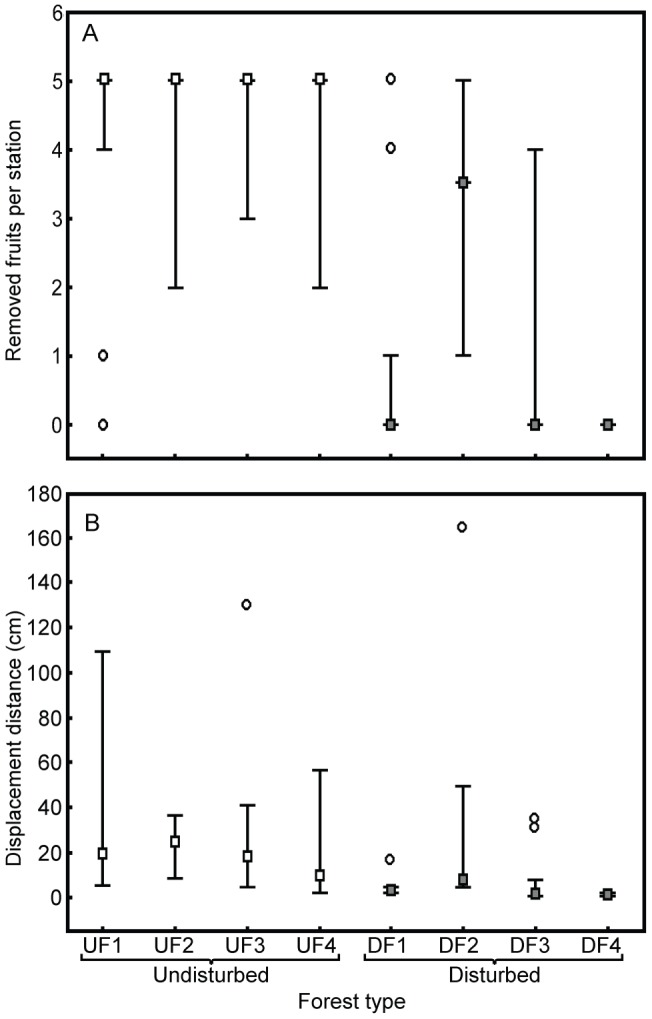
Dispersal of synthetic fruits by ants. (A) Removed synthetic fruits and (B) ‘fruit’ displacement distance in a fragmented Atlantic forest landscape. Experiments with synthetic fruits were carried out in two forest types: four undisturbed sites and four disturbed forest sites. Thirty stations were sampled per site. Each sampling station received five synthetic fruits at experiment beginning. Medians are represented by squares, while the lower and upper whiskers represent the 25% and 75% quartiles respectively. Outliers (values smaller or larger than 2 times the 75% minus the 25% quartiles) are represented by unfiled circles.

Patterns of ‘fruit’ discovery and exploitation by ants also differed between the two forest types. Synthetic fruits were discovered faster in undisturbed than in disturbed forest sites – after one hour, a higher number of the sampling stations had already been discovered by ants in undisturbed forest sites (29 to 30 attended stations per site) compared to disturbed forest fragments (24 to 27 stations; Wald's Z = −2.94, p<0.005). Moreover, undisturbed forest sites presented a higher number of stations with synthetic fruits removed by ants compared to disturbed sites (Wald's Z = −3.03, p<0.01; Figure 5B). At the end of the experiment the number of ‘fruits’ removed by ants per sampling station was higher at continuous than at fragmented forest sites (Wald's Z = −2.76, p<0.01), but there was also a considerable heterogeneity among sites (SD  = 1.44 was similar to the standard deviation observed for fixed effects, SD_UF_  = 1.45, and SD_DF_  = 2.11). Indeed, ‘fruit’ removal per station was more variable among disturbed forest sites than among undisturbed forest sites (Figure 6A).

Displacement distances ranged from 1 to 165 cm (n = 188 synthetic fruits). In general, synthetic fruits were displaced by ants to greater distances at undisturbed than at disturbed sites (Wald's Z = −2.79, p<0.01; Figure 6B). Again, there was a marked heterogeneity among the study sites (SD all sites  = 0.27; SD_UF_  = 0.29; SD_DF_  = 0.42).

Finally, the number of sampling stations with synthetic seeds entirely cleaned (i.e., ‘pulp’ detached) did not differ between the two forest types (Wald's Z = −1.18, p = 0.23; Figure 5B).

## Discussion

This novel experimental approach using ant-attractive synthetic fruits revealed that the deforestation process exerts a marked negative impact on the interaction between ants and fallen fruits on the ground of the Atlantic rainforest. Overall, undisturbed forest sites hosted a richer and distinct assemblage of ant species interacting with synthetic fruits than human-disturbed forest sites. Most importantly, in undisturbed sites we registered faster ‘fruit’ discovery by ants, higher numbers of ant species per sampling station, higher rates of ‘fruit’ removal by ants, and longer ‘fruit’ displacement distances than in the fragmented sites. Collectively, these results suggest that fallen fleshy fruits in disturbed forest fragments face a decreased probability of interacting with large ant species capable of offering dispersal services [17], [24], [25], [41], although a number of small species keep providing seed-cleaning services irrespective of forest type. Our study includes the Atlantic rainforest fragments into an expanding list of human-disturbed landscapes shown to negatively affect ant-fruit interactions [20], [31], [33], [34]. Even though replicates for the undisturbed forest sites were at least 1.5 km apart from one another, we concur that using only one continuous forest tract may limit far-reaching implications of our results (but see [50]).

### General Patterns Of Ant Attendance To Synthetic Fruits

Values of ant species richness at synthetic fruits (16 to 24 species per site) are quite similar to published results of ants attending fallen fleshy diaspores in other Atlantic rainforest sites: 26 and 16 ant species were recorded, respectively, at the lipid-rich arillate seeds of *Cabralea canjerana* (Meliaceae; [42]) and *Clusia criuva* (Clusiaceae; [41]), and 11 species attended the protein-rich fruits of *Guapira opposita* (Nyctaginaceae; [24]). Moreover, the spectrum of ant genera recorded at synthetic fruits is analogous to other Neotropical studies on the ant fauna interacting with fleshy diaspores of local floras [33], [43], [44], [45], [60], as well as to more general studies on Neotropical ground-dwelling ant communities [61], [62]. For instance, the genera *Pheidole* and *Solenopsis*, which are frequently recorded interacting with fleshy fruits (e.g. [43]; this study), are also among the most abundant and species-rich genera of tropical litter-dwelling ant communities [62], [63]. Indeed, the correspondence of the ant fauna consuming fleshy fruits with the overall ground-dwelling ant fauna confirms the generalized character of the interactions between ants and fallen fleshy diaspores [19].

The estimated species richness of ants at synthetic fruits was in general higher in undisturbed than in disturbed forest sites. Furthermore, we detected a significant difference in species composition between the two forest types. These patterns are not surprising given that many studies in tropical and non-tropical ecosystems report lower species richness in the ground-dwelling ant fauna of human-disturbed areas than in control sites [40], [64], [65], [66] and, most importantly, changes in ant species composition between disturbed and unidisturbed forest sites [28], [38], [39], [65]. In addition, because sampling stations with synthetic fruits were discovered faster and by a higher number of ant species in continuous forest sites than in disturbed forest fragments, we may predict that naturally-fallen fruits in undisturbed forests will face increased chances of interacting with an ant species capable of providing beneficial services such as seed-cleaning (e.g. [21], [22]), and/or directed dispersal to a soil-enriched nest microsite (e.g. [17], [24], [25]). A higher number of ant species per sampling point in less disturbed areas was also previously recorded by other studies [37], [66] (but see [65]).

### Frequency And Behaviour Of Particular Ant Groups

The observed higher occurrence of large ponerines (*Pachycondyla* and *Odontomachus*) in the undisturbed forest likely explains the higher scores of fruit removal rates and removal distances at this type of habitat. Large ponerines are especially important from a plant standpoint because an individual forager is capable of transporting small- to medium-sized diaspores (weight <1 g, *sensu* [53]) to relatively large distances (≈10 m), and thus act as an effective seed disperser. Actually, dispersal values may be higher than reported here since distances are frequently underestimated due to rapid disappearance of seed-carrying ponerines in the leaf litter [24], [41], [42]. The decreased abundance of these large ponerines in the studied Atlantic forest fragments, as well as their relevant role as seed dispersers, corroborates two recent studies in other Brazilian Biomes (Cerrado savanna, and semi-arid Caatinga) that are likewise subjected to diverse human-induced disturbances [20], [34]. In fact, large *Pachycondyla* and *Odontomachus* ants have been shown to be particularly sensitive to habitat fragmentation in the Atlantic forest [40].

Considering the prevalence of *Pheidole* ants regarding abundance and species richness in most tropical litter-dwelling ant faunas, and their mostly omnivorous habit [63], they seem less likely to be affected by forest fragmentation compared to other, more specialized ant taxa such as large ponerines (e.g. [20], [61], [65]). *Pheidole* species are commonly reported at fallen fleshy diaspores in tropical ecosystems (e.g. [17], [43]), sometimes acting as good seed removers due to their recruiting behaviour [20], [32]. Contrary to large ponerines, however, disturbance does not seem to negatively affect *Pheidole* ants [20], [32]. For instance, *Pheidole fallax* has been reported as the main seed remover at the edge of Costa Rican dry forests [32]. The presence of *Pheidole* ants may be crucial for the maintenance of ant services to non-myrmecochorous diaspores; as seen here, however, they do not completely substitute the large ponerines (see also [20]).

### Conclusions

Overall, our results on ants attending lipid-rich synthetic fruits parallel in many aspects the patterns previously described for interactions among ants and fallen fleshy fruits in the Atlantic forest [43], [44]. Thus, synthetic fruits proved useful at revealing patterns of ant attendance to fleshy fruits in variable ecological settings, and our data in particular enhance this novel method to better understand the ecology of ant-fruit interactions in a fragmented landscape. The adoption of this experimental approach in future studies should help circumvent practical problems in the field such as scarcity of fruits, or poor fruit quality resulting from infestation by insect larvae or fungal infection.

Most ant species were too small for transporting the synthetic fruits or even to entirely remove the pulp (Table S1), as also noted in other studies of ant-fruit interactions in tropical forests (e.g. [24], [42]). Thus ant-derived benefits to seeds and/or seedlings largely depend on the identity of the interacting ant species [17], [24], [41] (see also [67], [68] for true myrmecochores), whose frequency in turn may vary with the degree of habitat disturbance. Indeed, our experiment with synthetic fleshy fruits showed that the decreased occurrence of a particularly beneficial ant group (i.e., large ponerines) in the disturbed forest fragments corresponded with a decline in the ants' potential dispersal benefit to plants (i.e., less frequent dispersal and shorter displacement distance of ‘fruits’). The present study indicates that the deforestation process of the Atlantic rainforest (i.e., fragmentation and other human-induced disturbances) negatively affects the potentially mutualistic interaction between ground-dwelling ants and small to medium-sized fleshy fruits, corroborating other studies on ant-fruit interactions in human-disturbed habitats [20], [31], [33], [34]. The potential decrease in the benefits resulting from opportunistic ant-fruit interactions adds up to the already pessimistic scenario faced by vertebrate-dispersed plants [4], [11], [69], since frugivores are among the first to disappear from human-disturbed forest fragments [9], [10]. Our experimental results on ants as primary seed dispersers are thus particularly applicable for fragmented landscapes, since the frequency of fruits reaching the ground with the pulp still intact is expected to be higher in fragmented rather than in continuous forests. We hope that this study can contribute to reveal some of the consequences of the ongoing deforestation process of the Brazilian Atlantic rainforest to a relatively neglected part of the dispersal process of many zoochoric plants.

## Supporting Information

Figure S1 **Estimated richness of ant species attending lipid-rich synthetic fruits in four undisturbed sites within a continuous forest area (UFs) and in four disturbed forest fragments (DFs) in the Atlantic Forest (23°50**′**S, 47°20**′**W), municipalities of Piedade and Tapiraí, São Paulo State, southeast Brazil.**
(DOCX)

Table S1 **Ant species attending lipid-rich synthetic fruits in four sites within a undisturbed continuous forest area (UFs) and in four disturbed forest fragments (DFs) in the Atlantic Forest (23°50**′**S, 47°20**′**W), municipalities of Piedade and Tapiraí, São Paulo State, southeast Brazil.**
(DOC)

## References

[1] Fahrig L (2003) Effects of habitat fragmentation on biodiversity. Annual Review of Ecology Evolution and Systematics 34: 487–515.

[2] Laurance WF, Camargo JLC, Luizão RCC, Laurance SG, Pimm SL, et al (2011) The fate of Amazonian forest fragments: A 32-year investigation. Biological Conservation 144: 56–67.

[3] Aizen MA, Feinsinger P (1994) Habitat fragmentation, native insect pollinators, and feral honey bees in Argentine ‘Chaco Serrano’. Ecological Applications 4: 378–392.

[4] Cordeiro NJ, Howe HF (2003) Forest fragmentation severs mutualism between seed dispersers and an endemic African tree. Proceedings of the National Academy of Sciences of the United States of America 100: 14052–14056. 14614145 10.1073/pnas.2331023100 PMC283544

[5] Tylianakis JM, Tscharntke T, Lewis OT (2007) Habitat modification alters the structure of tropical host-parasitoid food webs. Nature 445: 202–205. 17215842 10.1038/nature05429

[6] Wilcock C, Neiland R (2002) Pollination failure in plants: why it happens and when it matters. Trends in Plant Science 7: 270–277. 12049924 10.1016/s1360-1385(02)02258-6

[7] Terborgh J, Nun?ez-Iturri G, Pitman NCA, Valverde FHC, Alvarez P, et al (2008) Tree recruitment in an empty forest. Ecology 89: 1757–1768. 18589539 10.1890/07-0479.1

[8] Howe HF, Smallwood J (1982) Ecology of seed dispersal. Annual Review of Ecology and Systematics 13: 201–228.

[9] Redford K (1992) The empty forest. BioScience 42: 412–422.

[10] Michalski F, Peres CA (2005) Anthropogenic determinants of primate and carnivore local extinctions in a fragmented forest landscape of southern Amazonia. Biological Conservation 124: 383–396.

[11] Silva JMC, Tabarelli M (2000) Tree species impoverishment and the future flora of the Atlantic forest of northeast Brazil. Nature 404: 72–74. 10716443 10.1038/35003563

[12] Cramer JM, Mesquita RCG, Williamson GB (2007) Forest fragmentation differentially affects seed dispersal of large and small-seeded tropical trees. Biological Conservation 137: 415–423.

[13] Chapman CA, Chapman LJ (1995) Survival without dispersers – Seedling recruitment under parents. Conservation Biology 9: 675–678.

[14] Dausmann KH, Glos J, Linsenmair KE, Ganzhorn JU (2008) Improved recruitment of a lemur-dispersed tree in Malagasy dry forests after the demise of vertebrates in forest fragments. Oecologia 157: 307–316. 18523808 10.1007/s00442-008-1070-6

[15] Frankie GW, Baker HG, Opler PA (1974) Comparative phenological studies of trees in tropical wet and dry forests in the lowlands of Costa Rica. Journal of Ecology 62: 881–919.

[16] Christianini AV, Oliveira PS (2009) The relevance of ants as seed rescuers of a primarily bird-dispersed tree in the Neotropical cerrado savanna. Oecologia 160: 735–745. 19399521 10.1007/s00442-009-1349-2

[17] Christianini AV, Oliveira PS (2010) Birds and ants provide complementary seed dispersal in a neotropical savanna. Journal of Ecology 98: 573–582.

[18] Vander Wall SB, Longland WS (2004) Diplochory: are two seed dispersers better than one? Trends in Ecology & Evolution 19: 155–161. 16701247 10.1016/j.tree.2003.12.004

[19] Rico-Gray V, Oliveira PS (2007) The ecology and evolution of ant-plant interactions. Chicago: The University of Chicago Press. 331 p.

[20] Christianini AV, Oliveira PS (2013) Edge effects decrease ant-derived benefits to seedlings in a neotropical savanna. Arthropod-Plant Interactions 7: 191–199.

[21] Oliveira PS, Galetti M, Pedroni F, Morellato LPC (1995) Seed cleaning by *Mycocepurus goeldii* ants (Attini) facilitates germination in *Hymenaea courbaril* (Caesalpiniaceae). Biotropica 27: 518–522.

[22] Leal IR, Oliveira PS (1998) Interactions between fungus-growing ants (Attini), fruits and seeds in cerrado vegetation in southeast Brazil. Biotropica 30: 170–178.

[23] Ohkawara K, Akino T (2005) Seed cleaning behavior by tropical ants and its anti-fungal effect. Journal of Ethology 23: 93–98.

[24] Passos L, Oliveira PS (2004) Interaction between ants and fruits of *Guapira opposita* (Nyctaginaceae) in a Brazilian sandy plain rainforest: ant effects on seeds and seedlings. Oecologia 139: 376–382. 15034779 10.1007/s00442-004-1531-5

[25] Böhning-Gaese K, Gaese BH, Rabemanantsoa SB (1999) Importance of primary and secondary seed dispersal in the Malagasy tree *Commiphora guillaumini* . Ecology 80: 821–832.

[26] Didham RK, Ghazoul J, Stork NE, Davis AJ (1996) Insects in fragmented forests: a functional approach. Trends in Ecology & Evolution 11: 255–260. 21237834 10.1016/0169-5347(96)20047-3

[27] Fowler HG, Pagani MI, da Silva OA, Forti LC, Saes NB (1989) A pest is a pest is a pest? The dilemma of neotropical leaf-cutting ants: keystone taxa of natural ecosystems. Environmental Management 13: 671–675.

[28] Crist TO (2009) Biodiversity, species interactions, and functional roles of ants (Hymenoptera: Formicidae) in fragmented landscapes: a review. Myrmecological News 12: 3–13.

[29] Diamond SE, Sorger DM, Hulcr J, Pelini SL, Del Toro I, Hirsch C, Oberg E, Dunn RR (2012) Who likes it hot? A global analysis of the climatic, ecological, and evolutionary determinants of warming tolerance in ants. Global Change Biology 18: 448–456.

[30] Bruna EM, Vasconcelos HL, Heredia S (2005) The effect of habitat fragmentation on communities of mutualists: Amazonian ants and their host plants. Biological Conservation 124: 209–216.

[31] Guimarães PR, Cogni R (2002) Seed cleaning of *Cupania vernalis* (Sapindaceae) by ants: edge effect in a highland forest in south-east Brazil. Journal of Tropical Ecology 18: 303–307.

[32] Zelikova TJ, Breed MD (2008) Effects of habitat disturbance on ant community composition and seed dispersal by ants in a tropical dry forest in Costa Rica. Journal of Tropical Ecology 24: 309–316.

[33] Zwiener VP, Bihn JH, Marques MCM (2012) Ant-diaspore interactions during secondary succession in the Atlantic forest of Brazil. Revista de Biologia Tropical 60: 933–942. 23894957 10.15517/rbt.v60i2.4028

[34] Leal LC, Andersen AN, Leal IR (2013) Anthropogenic disturbance reduces seed-dispersal services for myrmecochorous plants in the Brazilian Caatinga. Oecologia: In press. DOI: 10.1007/s00442-013-2740-6 10.1007/s00442-013-2740-6 23897500

[35] Mittermeier RA, Da Fonseca GAB, Rylands AB, Brandon K (2005) A brief history of biodiversity conservation in Brazil. Conservation Biology 19: 601–607.

[36] Tabarelli M, Aguiar AV, Ribeiro MC, Metzger JP, Peres CA (2010) Prospects for biodiversity conservation in the Atlantic Forest: Lessons from aging human-modified landscapes. Biological Conservation 143: 2328–2340.

[37] Ribeiro MC, Metzger JP, Martensen AC, Ponzoni FJ, Hirota MM (2009) The Brazilian Atlantic Forest: How much is left, and how is the remaining forest distributed? Implications for conservation. Biological Conservation 142: 1141–1153.

[38] Sobrinho TG, Schoereder JH (2007) Edge and shape effects on ant (Hymenoptera: Formicidae) species richness and composition in forest fragments. Biodiversity and Conservation 16: 1459–1470.

[39] Silva RR, Feitosa RSM, Eberhardt F (2007) Reduced ant diversity along a habitat regeneration gradient in the southern Brazilian Atlantic Forest. Forest Ecology and Management 240: 61–69.

[40] Leal IR, Filgueiras BKC, Gomes JP, Iannuzzi L, Andersen AN (2012) Effects of habitat fragmentation on ant richness and functional composition in Brazilian Atlantic forest. Biodiversity and Conservation 21: 1687–1701.

[41] Passos L, Oliveira PS (2002) Ants affect the distribution and performance of seedlings of *Clusia criuva*, a primarily bird-dispersed rain forest tree. Journal of Ecology 90: 517–528.

[42] Pizo MA, Oliveira PS (1998) Interaction between ants and seeds of a nonmyrmecochorous neotropical tree, *Cabralea canjerana* (Meliaceae), in the Atlantic forest of southeast Brazil. American Journal of Botany 85: 669–674. 21684948

[43] Pizo MA, Oliveira PS (2000) The use of fruits and seeds by ants in the Atlantic forest of southeast Brazil. Biotropica 32: 851–861.

[44] Passos L, Oliveira PS (2003) Interactions between ants, fruits and seeds in a restinga forest in south-eastern Brazil. Journal of Tropical Ecology 19: 261–270.

[45] Bieber AGD (2012) A fragmentação florestal e a interação entre formigas e diásporos carnosos na Floresta Atlântica [Doctor thesis]. Campinas, Brasil: Universidade Estadual de Campinas. 202 p.

[46] Oliveira-Filho AT, Fontes MAL (2000) Patterns of floristic differentiation among Atlantic forest in Southrastern Brazil and the influence of climate. Biotropica 32: 793–810.

[47] Banks-Leite C, Ewers RM, Pimentel RG, Metzger JP (2012) Decisions on temporal sampling protocol influence the detection of ecological patterns. Biotropica 44: 378–385.

[48] Lira PK, Tambosi LR, Ewers RM, Metzger JP (2012) Land-use and land-cover change in Atlantic Forest landscapes. Forest Ecology and Management 278: 80–89.

[49] Banks-Leite C, Ewers RM, Metzger JP (2010) Edge effects as the principal cause of area effects on birds in fragmented secondary forest. Oikos 119: 918–926.

[50] Uehara-Prado M, Brown KS, Freitas AVL (2007) Species richness, composition and abundance of fruit-feeding butterflies in the Brazilian Atlantic Forest: comparison between a fragmented and a continuous landscape. Global Ecology and Biogeography 16: 43–54.

[51] Alves-Costa CP, Lopes AV (2001) Using artificial fruits to evaluate fruit selection by birds in the field. Biotropica 33: 713–717.

[52] Bieber AGD, Silva PSD, Oliveira PS (2013) Attractiveness of fallen fleshy fruits to ants depends on previous handling by frugivores. Ecoscience 20: 85–89.

[53] Pizo MA, Oliveira PS (2001) Size and lipid content of nonmyrmecochorous diaspores: effects on the interaction with litter-foraging ants in the Atlantic rain forest of Brazil. Plant Ecology 157: 37–52.

[54] Raimundo RLG, Guimaraes PR, Almeida-Neto M, Pizo MA (2004) The influence of fruit morphology and habitat structure on ant-seed interactions: A study with artificial fruits. Sociobiology 44: 261–270.

[55] Hölldobler B, Wilson EO (1990) The ants. Massachusetts: Harvard University Press, Cambridge. 732 p.

[56] Krebs CJ (1998) Ecological methodology. Menlo Park, California: Addison Wesley Longman. 620 p.

[57] R Development Core Team (2011) R: A Language and Environment for Statistical Computing. Vienna, Austria: R Foundation for Statistical Computing. URL: http://www.R-project.org/.

[58] Sokal RR, Rohlf SR (1995) Biometry. New York, USA: WH Freeman. 887 p.

[59] Zuur A, Ieno E, Walker N, Saveliev A, Smith G (2009) Mixed effects models and extensions in ecology with R. New York: Springer. 574 p.

[60] Christianini AV, Mayhe-Nunes AJ, Oliveira PS (2012) Exploitation of fallen diaspores by ants: are there ant-plant partner choices? Biotropica 44: 360–367.

[61] Vasconcelos HL, Vilhena JMS, Caliri GJA (2000) Responses of ants to selective logging of a central Amazonian forest. Journal of Applied Ecology 37: 508–514.

[62] Ward PS (2000) Broad-scale patterns of diversity in leaf litter ant communities. In: Agosti D, Majer JD, Alonso LE, Schultz T, editors. Standard methods for measuring and monitoring biodiversity. Washington, D.C.: Smithsonian Institution Press. pp. 99–121.

[63] Wilson EO (2003) *Pheidole* in the New World: a dominant, hyperdiverse ant genus. Cambridge, MA.: Harvard University Press. 794 p.

[64] Suarez AV, Bolger DT, Case TJ (1998) Effects of fragmentation and invasion on native ant communities in coastal southern California. Ecology 79: 2041–2056.

[65] Vasconcelos HL (1999) Effects of forest disturbance on the structure of ground-foraging ant communities in central Amazonia. Biodiversity and Conservation 8: 409–420.

[66] Brühl CA, Eltz T, Linsenmair KE (2003) Size does matter–effects of tropical rainforest fragmentation on the leaf litter ant community in Sabah, Malaysia. Biodiversity and Conservation 12: 1371–1389.

[67] Horvitz CC, Beattie AJ (1980) Ant dispersal of *Calathea* (Marantaceae) seeds by carnivorous ponerines (Formicidae) in a tropical rain forest. American Journal of Botany 67: 321–326.

[68] Andersen AN, Morrison SC (1998) Myrmecochory in Australia's seasonal tropics: Effects of disturbance on distance dispersal. Australian Journal of Ecology 23: 483–491.

[69] Markl JS, Schleuning M, Forget PM, Jordano P, Lambert JE, et al (2012) Meta-analysis of the effects of human disturbance on seed dispersal by animals. Conservation Biology 26: 1072–1081. 22971077 10.1111/j.1523-1739.2012.01927.x

