# Supplementary material for: Assessing the Impact of Deforestation of the Atlantic Rainforest on Ant-Fruit Interactions: A Field Experiment Using Synthetic Fruits
Source: PLoS One. 2014 Feb 26;9(2):e90369. doi: 10.1371/journal.pone.0090369 (PMC3936012; doi:10.1371/journal.pone.0090369)

**Supporting information**

**Assessing the Impact of Deforestation of the Atlantic Rainforest on Ant-Fruit Interactions: A Field Experiment Using Synthetic Fruits**

Ana Gabriela D. Bieber, Paulo S. D. Silva, Sebastián F. Sendoya & Paulo S. Oliveira

**Figure S1. Estimated richness of ant species attending lipid-rich synthetic fruits in four undisturbed sites within a continuous forest area (UFs) and in four disturbed forest fragments (DFs) in the Atlantic Forest (23°50'S, 47°20'W), municipalities of Piedade and Tapiraí, São Paulo State, southeast Brazil.** **Mean richness and the 95% confidence intervals were calculated based on the Jackknife I estimation procedure. Lipid-rich synthetic fruits were exposed to ant visitation in 30 sampling stations per site.**


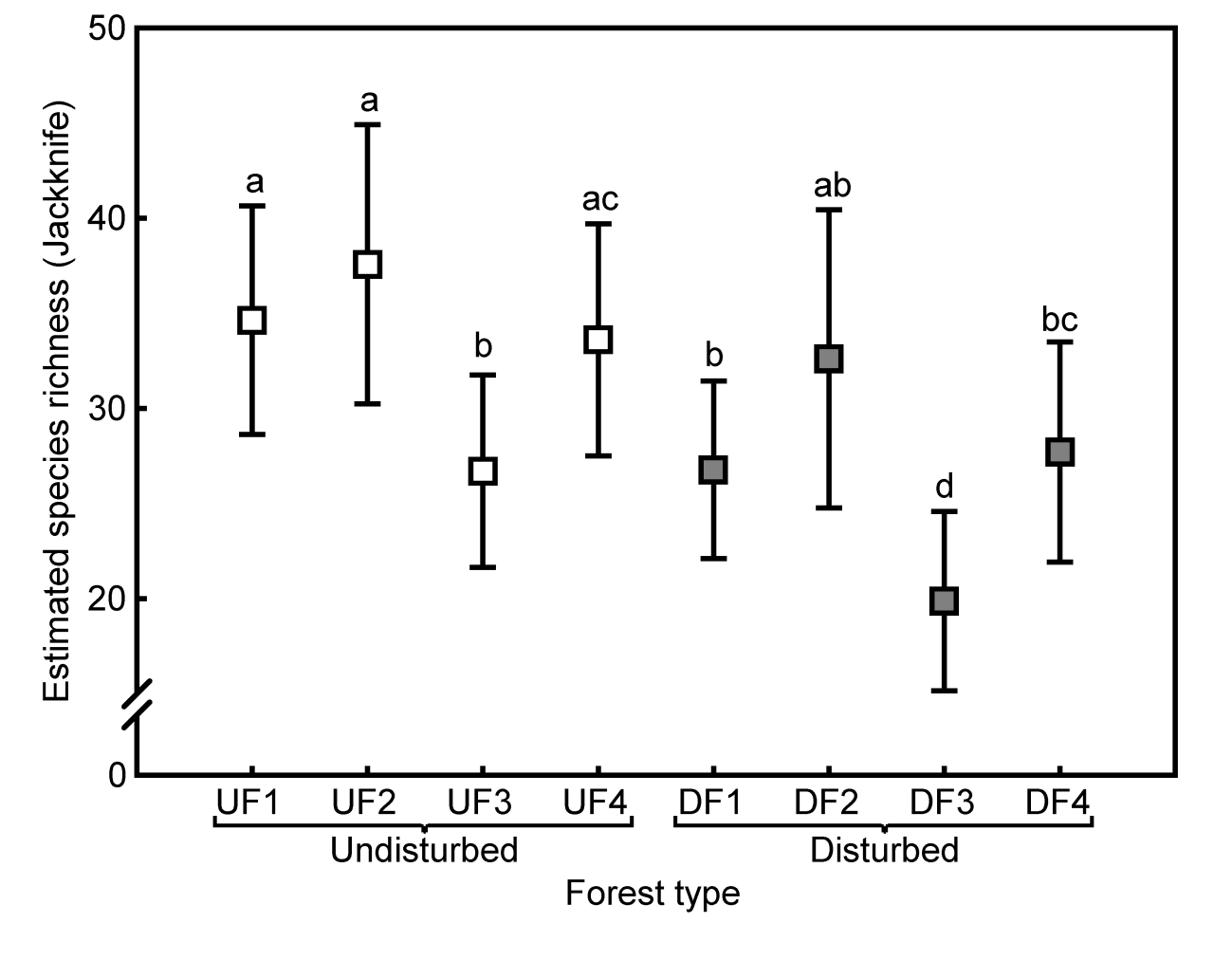

Supplement: Figure S1 — Estimated richness of ant species attending lipid-rich synthetic fruits in four undisturbed sites within a continuous forest area (UFs) and in four disturbed forest fragments (DFs) in the Atlantic Forest (23°50′S, 47°20′W), municipalities of Piedade and Tapiraí, São Paulo State, southeast Brazil. (DOCX) [file pone.0090369.s001.docx]
