# Supplementary material for: Assessing the Impact of Deforestation of the Atlantic Rainforest on Ant-Fruit Interactions: A Field Experiment Using Synthetic Fruits
Source: PLoS One. 2014 Feb 26;9(2):e90369. doi: 10.1371/journal.pone.0090369 (PMC3936012; doi:10.1371/journal.pone.0090369)
Supplement: Table S1 — Ant species attending lipid-rich synthetic fruits in four sites within a undisturbed continuous forest area (UFs) and in four disturbed forest fragments (DFs) in the Atlantic Forest (23°50′S, 47°20′W), municipalities of Piedade and Tapiraí, São Paulo State, southeast Brazil. (DOC) [file pone.0090369.s002.doc]

**Supporting information**

**Assessing the Impact of Deforestation of the Atlantic Rainforest on Ant-Fruit Interactions: A Field Experiment Using Synthetic Fruits**

Ana Gabriela D. Bieber, Paulo S. D. Silva, Sebastián F. Sendoya & Paulo S. Oliveira

T**able S1.** **List of ant species attending lipid-rich synthetic fruits in four undisturbed sites within a continuous forest area (UFs) and in four disturbed forest fragments (DFs) in the Atlantic Forest (23°50'S, 47°20'W), municipalities of Piedade and Tapiraí, São Paulo State, southeast Brazil.**

| **Subfamily** | **Species** | **Behaviour^#^** | **Continuous Forest Sites** | | | | | **Fragmented Forest Sites** | | | | | **Total Records** |
| --- | --- | --- | --- | --- | --- | --- | --- | --- | --- | --- | --- | --- | --- |
|  |  |  | **CF1** | **CF2** | **CF3** | **CF4** | **CFs total** | **FF1** | **FF2** | **FF3** | **FF4** | **FFs total** |  |
| Dolichoderinae | *Linepithema leucomelas* | RPC | 0 | 0 | 0 | 0 | 0 | 2 | 0 | 0 | 0 | 2 | 2 |
|  | *Linepithema pulex* | RPC | 0 | 0 | 0 | 0 | 0 | 0 | 0 | 11 | 0 | 11 | 11 |
|  | *Linepithema* sp. 4 | RPC | 0 | 0 | 0 | 0 | 0 | 1 | 0 | 0 | 0 | 1 | 1 |
| Ectatomminae | *Ectatomma edentatum* | RC, TR | 0 | 1 | 0 | 0 | 1 | 0 | 0 | 0 | 0 | 0 | 1 |
|  | *Gnamptogenys striatula* | RPC, TR | 1 | 2 | 1 | 0 | 4 | 0 | 0 | 4 | 0 | 4 | 8 |
| Formicinae | *Brachymyrmex* sp. 1 | RR, RC | 0 | 1 | 0 | 1 | 2 | 0 | 0 | 0 | 0 | 0 | 2 |
|  | *Brachymyrmex* sp. 2 | RPC | 0 | 1 | 0 | 0 | 1 | 1 | 0 | 0 | 0 | 1 | 2 |
|  | *Brachymyrmex* sp. 3 | RPC | 0 | 0 | 0 | 0 | 0 | 1 | 0 | 0 | 0 | 1 | 1 |
| Formicinae | *Brachymyrmex* sp. 4 | RPC, TR | 0 | 0 | 0 | 1 | 1 | 0 | 0 | 0 | 1 | 1 | 2 |
|  | *Nylanderia* sp. 2 | RPC | 1 | 0 | 0 | 0 | 1 | 3 | 1 | 0 | 2 | 6 | 7 |
| Heteroponerinae | *Heteroponera inermis* | RR | 0 | 0 | 0 | 0 | 0 | 0 | 1 | 0 | 0 | 1 | 1 |
| Myrmicinae | *Acromyrmex rugosus* | R | 1 | 0 | 0 | 0 | 1 | 0 | 0 | 0 | 0 | 0 | 1 |
|  | *Apterostigma* sp. 1 | RPC? | 0 | 1 | 0 | 0 | 1 | 0 | 0 | 0 | 0 | 0 | 1 |
|  | *Megalomyrmex iheringi* | RR, RC | 0 | 0 | 0 | 0 | 0 | 2 | 4 | 0 | 7 | 13 | 13 |
|  | *Oxyepoecus punctifrons* | RPC? | 0 | 1 | 0 | 0 | 1 | 0 | 0 | 0 | 0 | 0 | 1 |
|  | *Pheidole* sp. 1 | RPC | 3 | 9 | 11 | 7 | 30 | 1 | 3 | 0 | 5 | 9 | 39 |
|  | *Pheidole* sp. 2 | RC, TR | 2 | 1 | 0 | 1 | 4 | 0 | 0 | 0 | 0 | 0 | 4 |
|  | *Pheidole* sp. 3* | RPC, TR | 9 | 8 | 11 | 13 | 41 | 0 | 6 | 4 | 0 | 10 | 51 |
|  | *Pheidole* sp. 4 | RPC | 1 | 3 | 0 | 0 | 4 | 7 | 0 | 0 | 9 | 16 | 20 |
|  | *Pheidole* sp. 5 | RPC, TR | 0 | 3 | 4 | 1 | 8 | 0 | 1 | 1 | 2 | 4 | 12 |
|  | *Pheidole* sp. 6 | RPC, TR | 6 | 0 | 0 | 4 | 10 | 0 | 0 | 0 | 2 | 2 | 12 |
|  | *Pheidole* sp. 7* | RC, RR | 1 | 1 | 1 | 2 | 5 | 0 | 0 | 0 | 0 | 0 | 5 |
|  | *Pheidole* sp. 8* | RC, RR | 5 | 4 | 4 | 3 | 16 | 0 | 1 | 7 | 1 | 9 | 25 |
|  | *Pheidole* sp. 11 | RPC | 0 | 0 | 0 | 0 | 0 | 0 | 1 | 0 | 0 | 1 | 1 |
| Myrmicinae | *Pheidole* sp. 12* | RPC | 0 | 0 | 1 | 0 | 1 | 6 | 0 | 2 | 8 | 16 | 17 |
|  | *Pheidole* sp. 14 | RPC | 1 | 1 | 2 | 0 | 4 | 4 | 2 | 1 | 1 | 8 | 12 |
|  | *Pheidole* sp. 15 | RPC | 0 | 0 | 0 | 0 | 0 | 0 | 0 | 0 | 1 | 1 | 1 |
|  | *Pheidole* sp. 18* | RC, TR | 2 | 0 | 0 | 0 | 2 | 1 | 1 | 8 | 1 | 11 | 13 |
|  | *Pheidole* sp. 19* | RC, RR | 2 | 0 | 1 | 1 | 4 | 1 | 0 | 2 | 4 | 7 | 11 |
|  | *Pheidole* sp. 20* | RR | 0 | 0 | 0 | 1 | 1 | 0 | 3 | 3 | 0 | 6 | 7 |
|  | *Pheidole* sp. 24* | RPC | 0 | 0 | 0 | 0 | 0 | 4 | 0 | 0 | 1 | 5 | 5 |
|  | *Pheidole* sp. 27* | RR | 1 | 1 | 0 | 1 | 3 | 0 | 1 | 0 | 0 | 1 | 4 |
|  | *Pheidole* sp. 28 | RPC | 0 | 0 | 0 | 0 | 0 | 2 | 0 | 0 | 0 | 2 | 2 |
|  | *Pheidole* sp. 29 | RPC | 1 | 0 | 0 | 0 | 1 | 0 | 0 | 0 | 0 | 0 | 1 |
|  | *Solenopsis* sp. 1 | RPC | 5 | 7 | 1 | 1 | 14 | 0 | 0 | 0 | 0 | 0 | 14 |
|  | *Solenopsis* sp. 2 | RPC | 4 | 0 | 0 | 1 | 5 | 0 | 0 | 0 | 0 | 0 | 5 |
|  | *Solenopsis* sp. 3 | RPC | 0 | 2 | 0 | 0 | 2 | 0 | 0 | 0 | 1 | 1 | 3 |
|  | *Solenopsis* sp. 5 | RPC | 0 | 0 | 1 | 4 | 5 | 7 | 2 | 2 | 0 | 11 | 16 |
|  | *Solenopsis* sp. 7 | RPC | 6 | 5 | 4 | 3 | 18 | 5 | 1 | 8 | 12 | 26 | 44 |
|  | *Solenopsis* sp. 8 | RPC | 1 | 1 | 1 | 4 | 7 | 1 | 2 | 0 | 1 | 4 | 11 |
| Myrmicinae | *Solenopsis* sp. 9 | RPC | 1 | 2 | 0 | 1 | 4 | 2 | 1 | 1 | 0 | 4 | 8 |
|  | *Solenopsis* sp. 10 | RPC? | 0 | 0 | 0 | 1 | 1 | 0 | 0 | 0 | 0 | 0 | 1 |
|  | *Solenopsis* sp. 11 | RC, RR | 1 | 0 | 2 | 1 | 4 | 0 | 0 | 0 | 0 | 0 | 4 |
|  | *Solenopsis* sp. 12 | RPC | 0 | 0 | 1 | 0 | 1 | 0 | 0 | 0 | 0 | 0 | 1 |
|  | *Solenopsis* sp. 13 | RPC | 0 | 1 | 0 | 0 | 1 | 2 | 1 | 1 | 3 | 7 | 8 |
|  | *Wasmannia affinis* | RPC | 2 | 6 | 6 | 4 | 18 | 0 | 3 | 3 | 1 | 7 | 25 |
| Ponerinae | *Hypoponera* sp. 1 | RPC | 0 | 0 | 1 | 0 | 1 | 0 | 1 | 0 | 0 | 1 | 2 |
|  | *Hypoponera* sp. 3 | RPC | 0 | 1 | 0 | 0 | 1 | 0 | 0 | 0 | 0 | 0 | 1 |
|  | *Hypoponera* sp. 4 | RPC | 0 | 0 | 0 | 0 | 0 | 0 | 1 | 0 | 0 | 1 | 1 |
|  | *Odontomachus chelifer* | R | 3 | 2 | 0 | 0 | 5 | 0 | 0 | 0 | 0 | 0 | 5 |
|  | *Pachycondyla striata* | R | 6 | 3 | 5 | 10 | 24 | 2 | 8 | 2 | 0 | 12 | 36 |
|  | Species richness per site |  | 24 | 24 | 18 | 22 | 40 | 20 | 21 | 16 | 19 | 36 | 51 |

Values indicate the number of stations per site where each ant species was captured (N = 30 stations in each of the eight sites). Species authorities can be checked out at the website “Antbase” (edited by D. Agosti and N. F. Johnson, 2005; URL: http://www.antbase.org).

*Pheidole spp. considered large (worker length ≥ 3 mm).

^#^Key to ant behaviour: R = individual worker remove fruit (> 5 cm); RR =recruited workers (> 5 ants) remove fruit (> 5cm); TR = try to remove fruit (< 5 cm); RC = recruited workers (> 5 ants) clean the seed by continual removal of pulp on spot; RPC =recruited workers remove the pulp partially (max. 25%) on spot, normally subordinate species.
